# Supplementary material for: High-throughput mutagenesis identifies mutations and RNA-binding proteins controlling CD19 splicing and CART-19 therapy resistance
Source: Nat Commun. 2022 Sep 22;13:5570. doi: 10.1038/s41467-022-31818-y (PMC9500061; doi:10.1038/s41467-022-31818-y)
Supplement: Supplementary file 1 — Supplementary Information [file 41467_2022_31818_MOESM1_ESM.pdf]

High-throughput mutagenesis identifies mutations and RNA-binding proteins controlling *CD19* splicing and CART-19 therapy resistance

Mariela Cortés-López[1#], Laura Schulz[1#], Mihaela Enculescu[1#], Claudia Paret[2,3,4], Bea Spiekermann[1], Mathieu Quesnel-Vallières[5,6], Manuel Torres-Diz[7], Sebastian Unic[8], Anke Busch[1], Anna Orekhova[1], Monika Kuban[5], Mikhail Mesitov[1], Miriam M. Mulorz[1], Rawan Shraim[7,9], Fridolin Kielisch[1], Jörg Faber[2,3,4], Yoseph Barash[5,6], Andrei Thomas-Tikhonenko[7,10], Kathi Zarnack[11,12,*], Stefan Legewie[1,8,13,*], and Julian König[1,*]

[1] Institute of Molecular Biology (IMB), Ackermannweg 4, 55128 Mainz, Germany
[2] Department of Pediatric Hematology/Oncology, Center for Pediatric and Adolescent Medicine, University Medical Center of the Johannes Gutenberg University Mainz, 55131 Mainz, Germany
[3] University Cancer Center (UCT), University Medical Center of the Johannes Gutenberg University Mainz, 55131 Mainz, Germany
[4] German Cancer Consortium (DKTK), site Frankfurt/Mainz, Germany, German Cancer Research Center (DKFZ), 69120 Heidelberg, Germany
[5] Department of Genetics, Perelman School of Medicine at the University of Pennsylvania, Philadelphia, PA 19104, USA
[6] Department of Biochemistry and Biophysics, Perelman School of Medicine at the University of Pennsylvania, Philadelphia, PA 19104, USA
[7] Division of Cancer Pathobiology, Children's Hospital of Philadelphia, Philadelphia, PA 19104, USA
[8] Department of Systems Biology, Institute for Biomedical Genetics (IBMG), University of Stuttgart, Allmandring 30E, 70569 Stuttgart, Germany
[9] Department of Biomedical and Health Informatics, Children's Hospital of Philadelphia, University of Pennsylvania Perelman School of Medicine, Philadelphia, PA 19104, USA
[10] Department of Pathology & Laboratory Medicine, Perelman School of Medicine at the University of Pennsylvania, Philadelphia, PA 19104, USA
[11] Buchmann Institute for Molecular Life Sciences (BMLS), Max-von-Laue-Str. 15, 60438 Frankfurt, Germany
[12] Faculty Biological Sciences, Goethe University Frankfurt, Max-von-Laue-Str. 15, 60438 Frankfurt, Germany
[13] Stuttgart Research Center for Systems Biology (SRCSB), University of Stuttgart, Stuttgart, Germany

# These authors contributed equally.
* Corresponding authors: Kathi Zarnack (kathi.zarnack@bmls.de), Stefan Legewie (legewie@iig.uni-stuttgart.de), Julian König (j.koenig@imb-mainz.de)

SUPPLEMENTARY INFORMATION

Table of content:

Supplementary Figures 2
Supplementary Tables 17
Supplementary References 20

Supplementary Figures

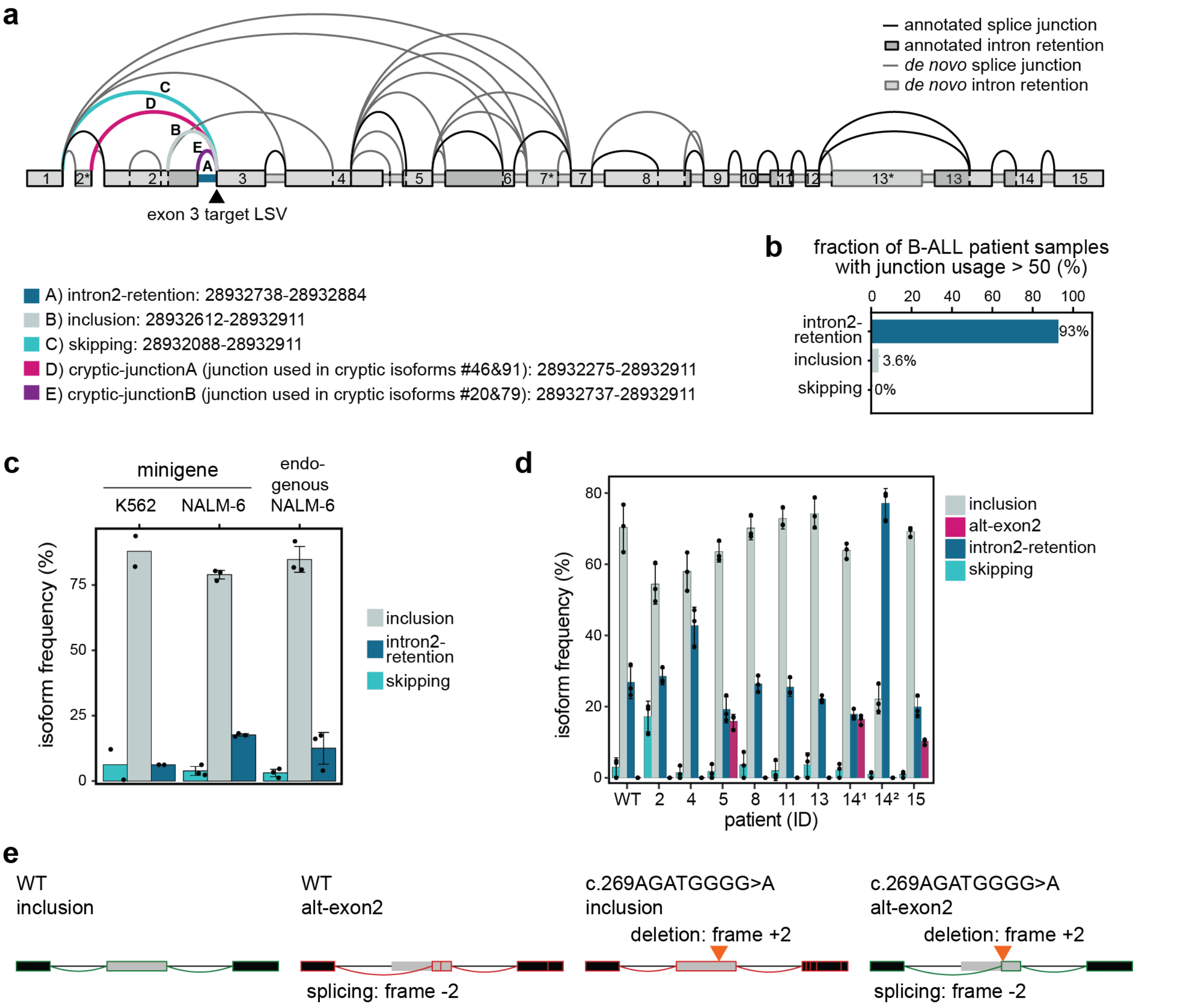

Supplementary Figure 1. *CD19* mis-splicing in TARGET B-ALL and Orlando datasets. (a) *CD19* shows extensive mis-splicing in B-ALL patients. Splice junctions were quantified with MAJIQ[1] for 220 B-ALL patients from the Therapeutically Applicable Research To Generate Effective Treatments (TARGET) programme (https://ocg.cancer.gov/programs/target). Splicegraph shows all splice junctions with a usage level (percent selected index, PSI) of at least 5% in any patient. Junctions and target exon of the local splicing variation (LSV) shown in (b) and Figure 1c, d are highlighted. **(b)** Intron 2 retention is the predominant isoform in B-ALL patients. Barchart quantifies the fraction of patients (220 B-ALL patients from the TARGET B-ALL programme) in which a given junction rises to PSI > 50%. **(c)** The minigene generates the same isoforms as the endogenous *CD19* gene in NALM-6 cells. Semiquantitative RT-PCR was performed to detect isoforms generated from exons 1-3 of the *CD19* minigene and the endogenous *CD19* gene in NALM-6 cells. Quantifications (mean and data points) of individual isoforms corresponding to Figure 1g. Error bars indicate standard deviation of mean (s.d.m.) if n > 2 replicates. **(d)** Patient mutations cause splicing changes in the *CD19* minigene. Semiquantitative RT-PCR as in (c) for minigene variants including nine mutations from B-ALL patients. Quantifications (mean and data points) of individual isoforms corresponding to Figure 1i. Patient ID numbers as reported in Orlando et al.[2]. 14.1 and 14.2 correspond to distinct mutations from patient #14. Error bars indicate s.d.m., n = 3 replicates. **(e)** The deletion c.269AGATGGGG>A from patient #5 in Orlando et al.[2] introduces a frameshift (+2) that is compensated by the activation of an out-of-frame cryptic splice site (-2). Shown are the major isoforms inclusion and alt-exon2 and their coding potential in the absence (left)

or presence (right) of the deletion (orange arrowhead). Schematic representation of depicts exons 1-3 (boxes) and introns (horizontal lines) with splice junctions for each isoform (arches). Colour indicates coding potential (green, coding; red, non-coding).

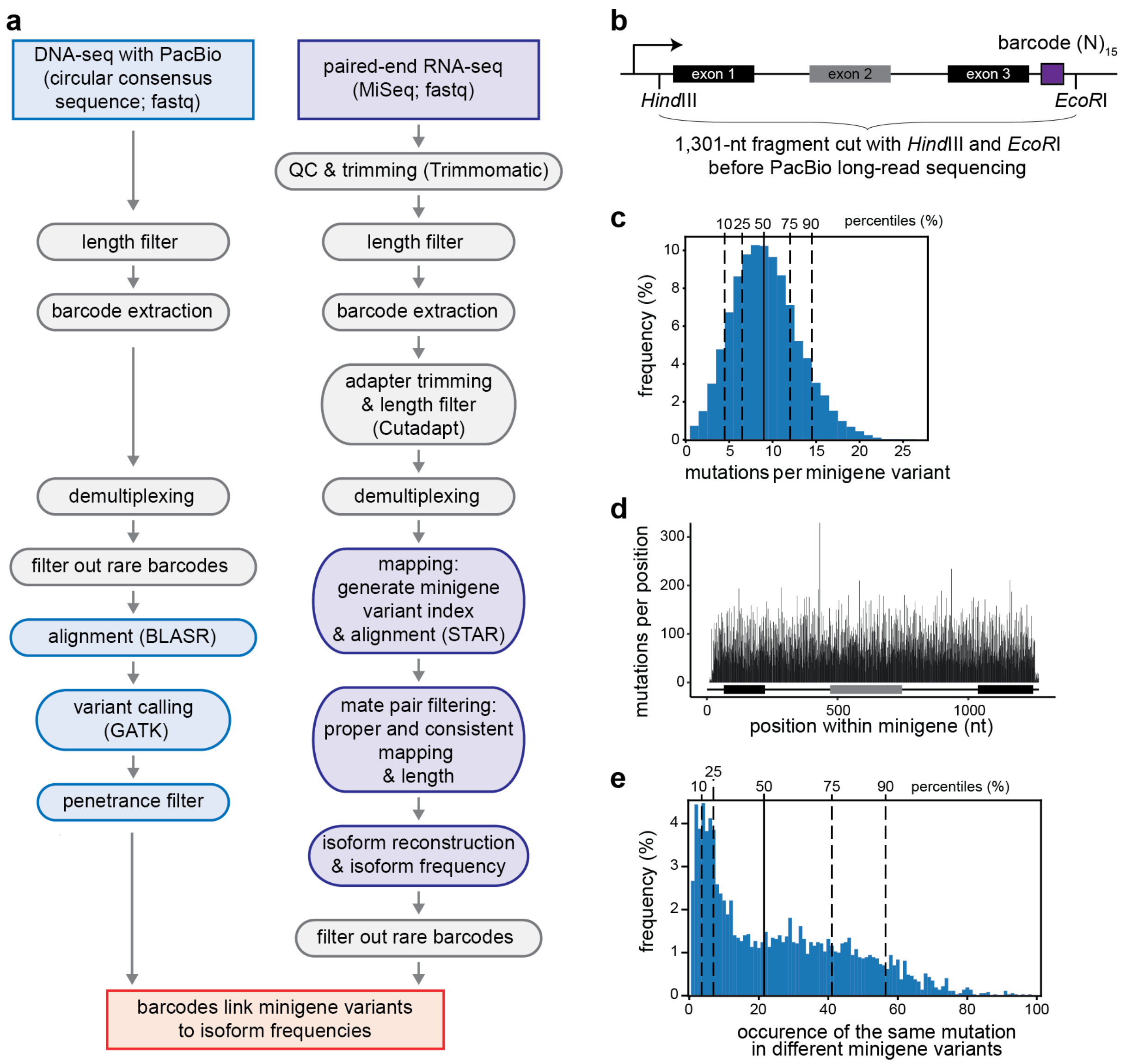

Supplementary Figure 2. Long-read sequencing identifies the introduced mutations. (a) Analysis pipeline for the targeted DNA-seq and RNA-seq data. Left: Long-read DNA-seq data (PacBio, Pacific Bioscience) in the form of circular consensus sequences (CSS) were filtered by length (1,150-1,500 nt). 15-nt barcodes were extracted and demultiplexed, keeping only minigenes supported by at least 4 CSS. Alignment to the minigene reference was performed with BLASR[3] and variants were called using GATK HaplotypeCaller[4]. Mutations in the minigene were filtered by the "penetrance score" (allele frequency, AF), discarding all the barcodes with more than 25% variants of low penetrance (AF < 0.8). Right: Short-read RNA-seq data (Illumina) were trimmed based on quality using Trimmomatic[5] and filtered by length (305 nt for read 1, 157 nt for read 2), and adapters were trimmed using Cutadapt[6] and 15-nt barcodes were extracted and demultiplexed, keeping only minigenes supported by at least 100 read pairs. Alignment to the specific mutated version of the minigene was performed using STAR[7]. Isoform reconstruction and isoform frequency estimation was done using custom scripts (see Methods). Only minigenes with 100 or more read pairs usable for isoform reconstruction were kept. **(b)** Structure of the *CD19* minigene fragment for long-read sequencing (PacBio) to identify introduced mutations. The minigene covers exons 1-3 with the intervening introns, followed by a 15-nt barcode. The fragment for PacBio sequencing is defined by the restriction sites for *Hind*III upstream of exon 1 and *EcoR*I downstream of the barcode sequence. **(c)** 91.6% of the minigene variants carry five or more mutations. Histogram shows number of mutations per minigene for 10,295 mutated minigene variants. **(d)** 4,255

distinct mutations are spread along the *CD19* minigene, with an average of 21 mutations per position. Barplot shows the sum of mutations per position in the minigene. **(e)** 81.9% of the mutations occur in at least three minigenes, which is sufficient for a reliable estimation of single mutation effects (Supplementary Figure 4c). Histogram shows the frequencies of the same mutations in different minigene variants.

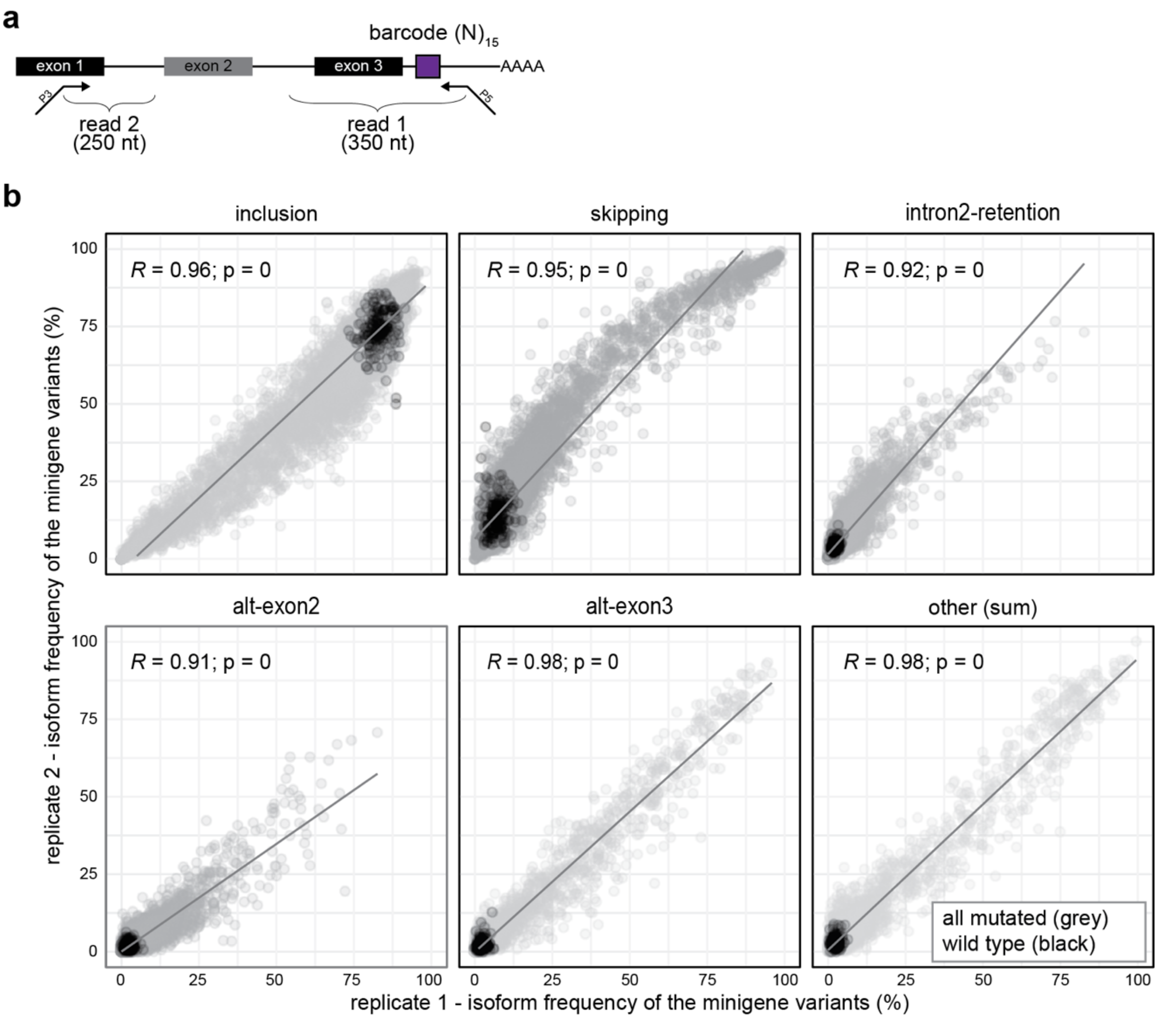

Supplementary Figure 3. Isoform measurements from targeted RNA-seq results are consistent between replicates. (a) Description of the short-read RNA-seq strategy (Illumina) to capture the splicing products in the *CD19* minigene. Read 2 (250 nt) extends beyond exon 1, i.e., covering the exon 1/exon 2 junction, while read 1 (350 nt) includes the 15-nt barcode and extends beyond exon 3. **(b)** The isoform measurements correlate well between replicates. Scatterplots compare isoform frequencies for five major isoforms as well as the sum of 96 cryptic isoforms between replicate 1 and 2. Each dot represents a particular minigene captured in both replicates. WT and mutated minigenes appear in black and grey, respectively. Pearson correlation coefficients (*R*) and associated *P* values (two-sided) are given.

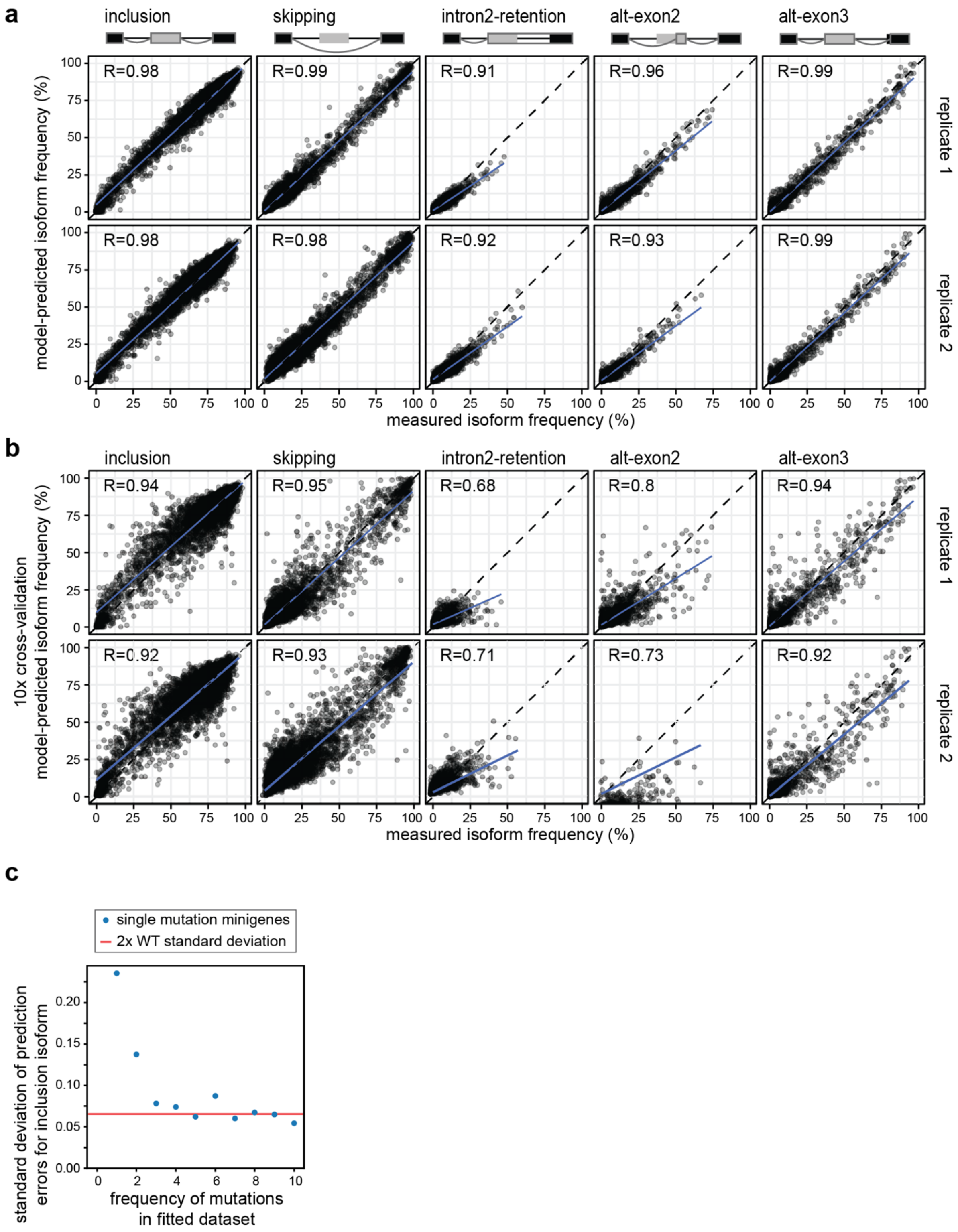

Supplementary Figure 4. The softmax regression model performs well for training and test data. (a) Regression model fits measured combined mutation effects (i.e., minigene measurements) with high accuracy. Scatterplots show frequencies of the five major isoforms in the measurements (x-axis) against the model fit (y-axis) for two biological replicates and 9,321 minigene variants used in model training. Pearson correlation coefficients (*R*) are shown for each scatterplot. **(b)** Cross-validation confirms the predictive power of the model for minigenes not used in training. The minigene library was randomly split into ten equally sized subsets. During 10-fold cross-validation, the softmax regression model was fitted to all data

excluding one subset. Scatterplots compare model-predicted splicing outcome for left-out subsets to corresponding experimental data for all major splice isoforms and are an overlay of the results of all cross-validation runs. Representation as in (a). **(c)** The model correctly infers single mutation effects. Seven single-mutation minigenes in which inclusion is significantly changed were left-out separately from softmax regression fitting and their effects were predicted based on the fit to the remaining minigene data. This procedure was repeated while additionally excluding random permutations of other minigenes containing the mutation. The standard deviation of the prediction error (y-axis) is plotted against the number of minigenes used in model training (x-axis). The inference power of the model reaches two standard deviations of the WT minigenes (horizontal line) if more than two minigenes containing the mutation are considered in model training. See Methods for details.

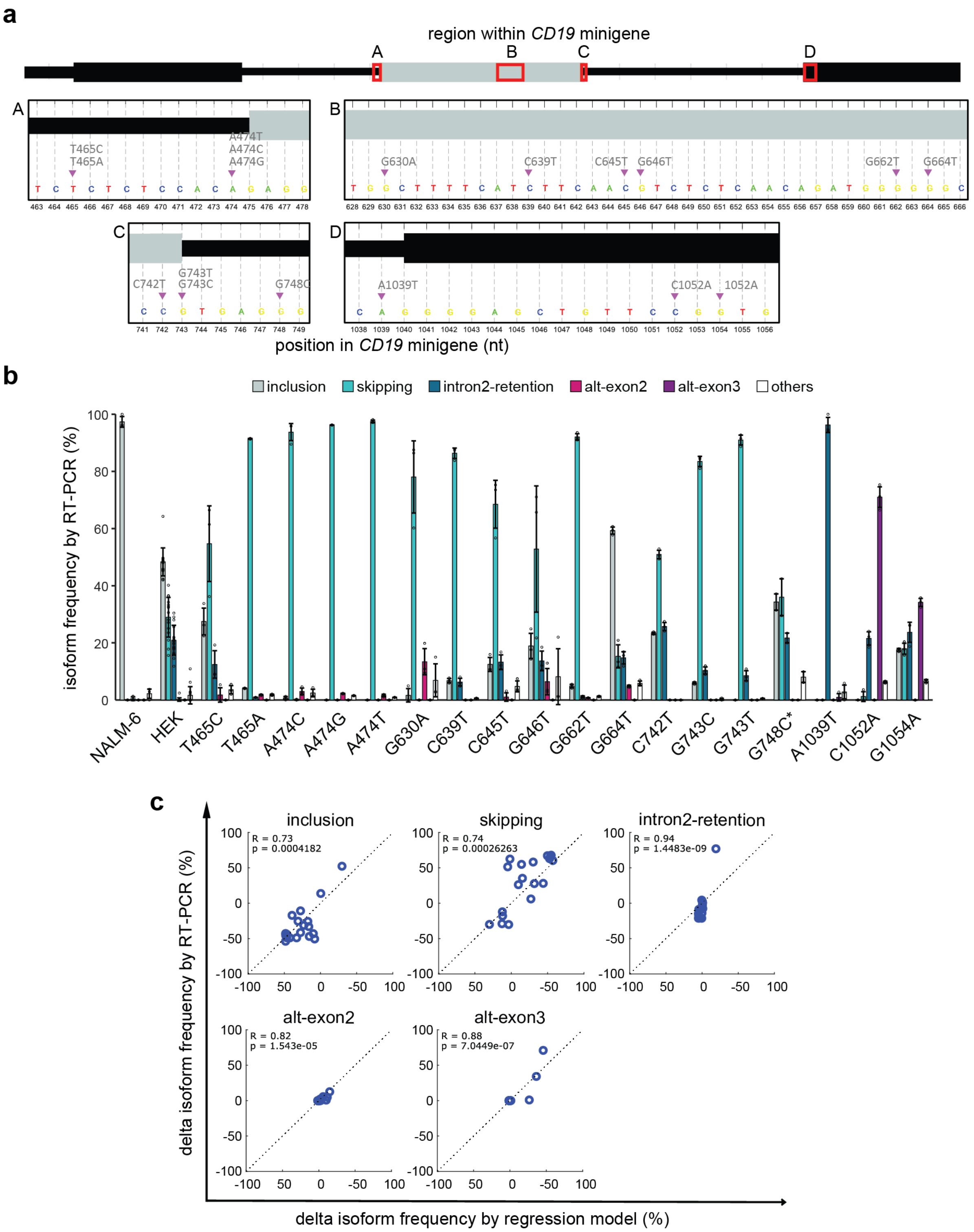

Supplementary Figure 5. RT-PCR measurements confirm the model predictions for 19 individual point mutations. (a) To test selected regression predictions, we generated 19 minigenes with individual point mutations that are predicted to affect at least one isoform (Supplementary Data 4). Point mutations were introduced by targeted mutagenesis. **(b)** Splicing outcome was quantified using RT-PCR followed by capillary electrophoresis. Quantifications (mean and data points) of individual isoforms corresponding to Figure 3e. 'NALM-6', splicing pattern of WT minigenes (RNA-seq) in the mutagenesis screen, 'HEK293', RT-PCR-based quantification of the baseline minigene containing mutation G742C in HEK293 cells. G748C* is a minigene containing G748C but lacking G742C. Error bars indicate

standard s.d.m. if n > 2 replicates. **(c)** Splicing patterns in response to single mutations correlate with regression predictions. Splicing outcomes from 19 *CD19* minigene variants containing single point mutations (y-axis) are related to single mutation predictions of the regression model (x-axis; mean of two fits, each explaining one mutagenesis replicate). Changes in the isoform frequency of the major isoforms are expressed as differences (delta) relative to the baseline. Pearson correlation coefficients and *P* values (two-sided) were calculated for each isoform (see Figure 3f for correlation over all isoforms).

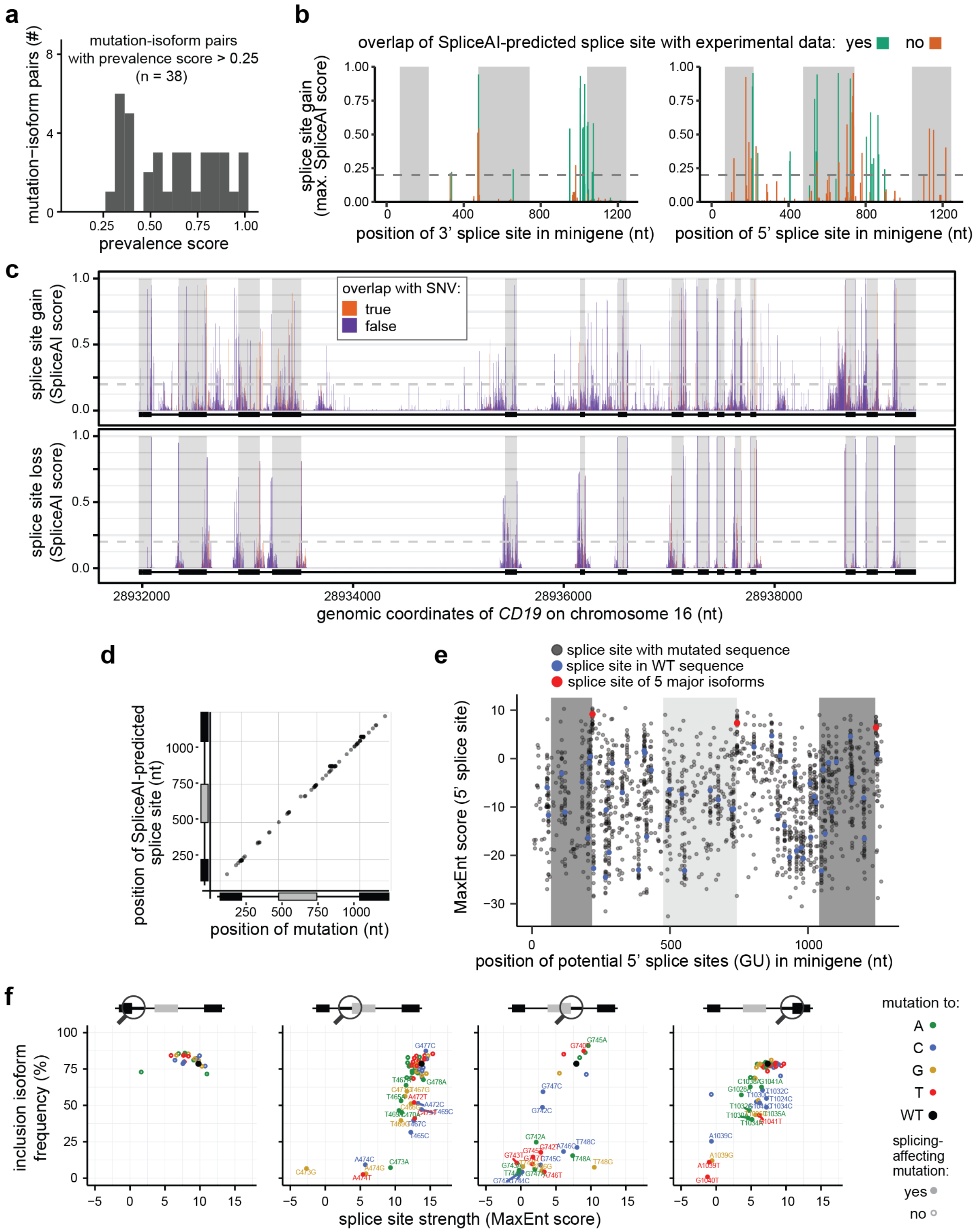

Supplementary Figure 6. Multiple mutations give rise to distinct cryptic isoforms. **(a)** Multiple mutations are associated with a specific cryptic isoform. Histogram shows distribution of prevalence scores for 38 mutation-isoform pairs (prevalence score > 0.25). A prevalence score of 1 indicates perfect correspondence between mutation and isoform. **(b)** SpliceAI[8] predictions for gained cryptic splice sites overlap with experimental data. Barplot shows the maximum SpliceAI score ("acceptor gain") for all the mutations that increase the probability of a given cryptic splice site to be used (38 mutations with Splice AI score [gain] > 0.5, including 15 and 23 gained 3' [left] and 5' splice sites [right]). Dotted horizontal line represents the recommended minimum threshold for a SpliceAI prediction (SpliceAI score >

0.2)[8]. Predicted gained splice sites that also appear in our experimental data are shown in green. **(c)** SpliceAI predicts splice-changing mutations across the full *CD19* gene locus. Barplots show the maximum SpliceAI score per position. Scores are separately shown for the gain (top) or loss (bottom) of splice sites. Colour code indicates overlap with reported variants (from gnomAD, ClinVar, COSMIC V94, Ensembl and TARGET B-ALL). 24 and 13 mutations reach a SpliceAI score > 0.2 for the gain and loss of splice sites, respectively (Supplementary Data 5). **(d)** SpliceAI-predicted splicing-affecting mutations reside on average within 6 nt from the cryptic splice site generated. Scatterplot shows location of the gained cryptic splice sites with respect to the mutations. Only the splice site with the highest score for each mutation is considered. **(e)** The 5' splice sites of the main isoforms (red) are stronger than most other 5' splice sites in the *CD19* minigene sequence. Dotplot shows splice site strengths (MaxEnt score)[9] for putative 5' splice sites in WT (blue) and mutated (grey) minigenes in a 9-nt sliding window containing a GU dinucleotide at positions 4-5. 5' splice sites used in the five major isoforms are shown in red. **(f)** Mutation effects at 3' and 5' splice sites of *CD19* exons 2 and 3 are consistent with predicted splice site strengths. Mutations are coloured according to the changed nucleotides. Scores for WT sequence are coloured in black. Splicing-affecting mutations (according to our results) are shown as filled circles and labelled.

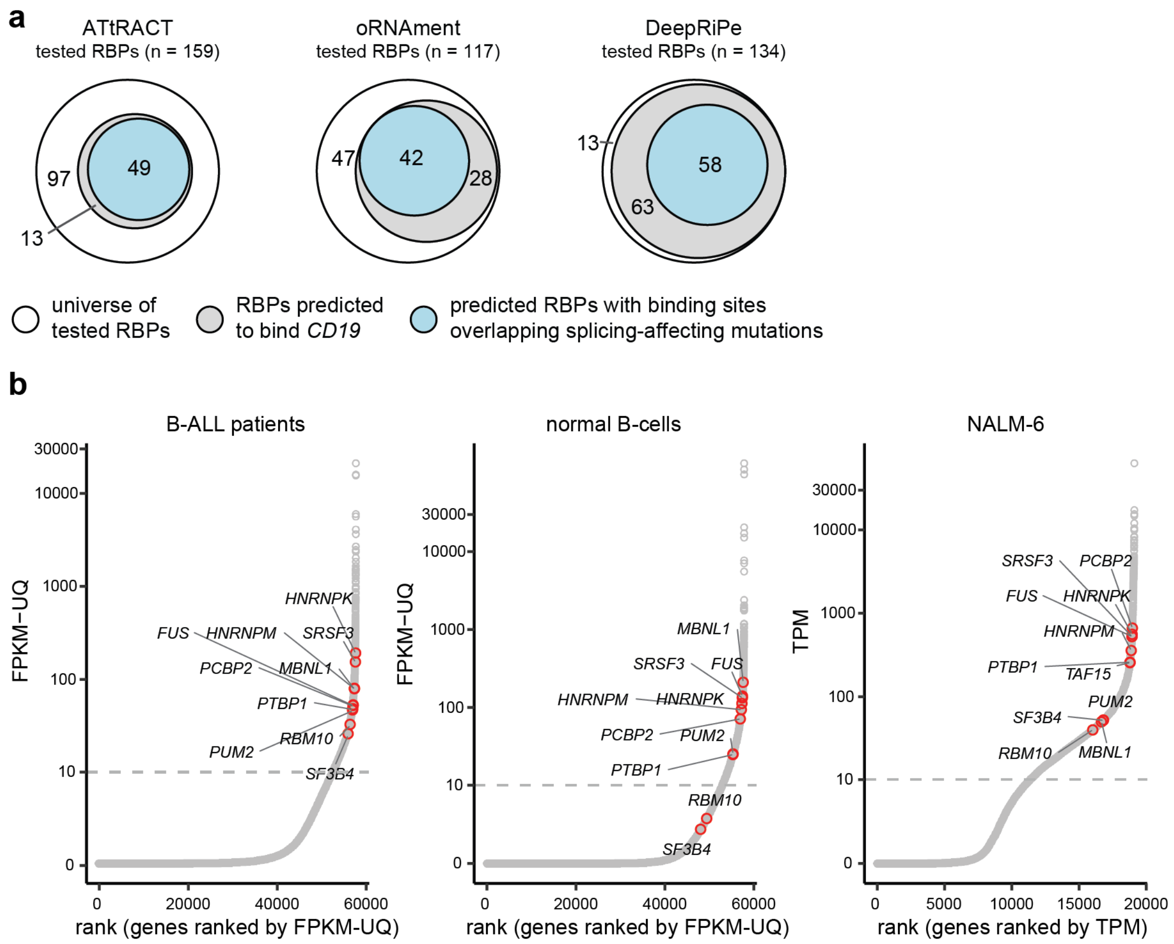

Supplementary Figure 7. *In silico* RBP binding site predictions suggest dozens of candidate regulators of *CD19* alternative splicing. (a) *In silico* predictions of RBP binding sites were performed with ATtRACT[10] and oRNAment[11] as well as of point mutations affecting RBP binding using DeepRiPe[12]. For each prediction tool, the total number of available RBPs (white circles) is split up into those that are predicted to bind *CD19* (grey circles) and whose predicted binding sites overlap with splicing-affecting mutations from our data (blue circles). Numbers refer to exclusive RBPs in each area. **(b)** Predicted RBPs were filtered based on their mean expression observed in B-ALL patients reported in[13]. Plot shows ranked mean expression values for all detected genes in samples from B-ALL patients (n = 57,773 genes, 1,988 patients), normal B-cells[14] (n = 57,773 genes, 147 samples) and NALM-6 cells[15] (n = 19,110 genes, 1 sample). Highlighted in red are the RBP candidate genes (n = 11) tested in knockdown experiments. TPM, transcripts per million. FPKM-UQ, fragments per kilobase of transcript per million mapped reads upper quartile, a modified RNA-seq normalisation method (https://docs.gdc.cancer.gov/Encyclopedia/pages/HTSeq-FPKM-UQ/).

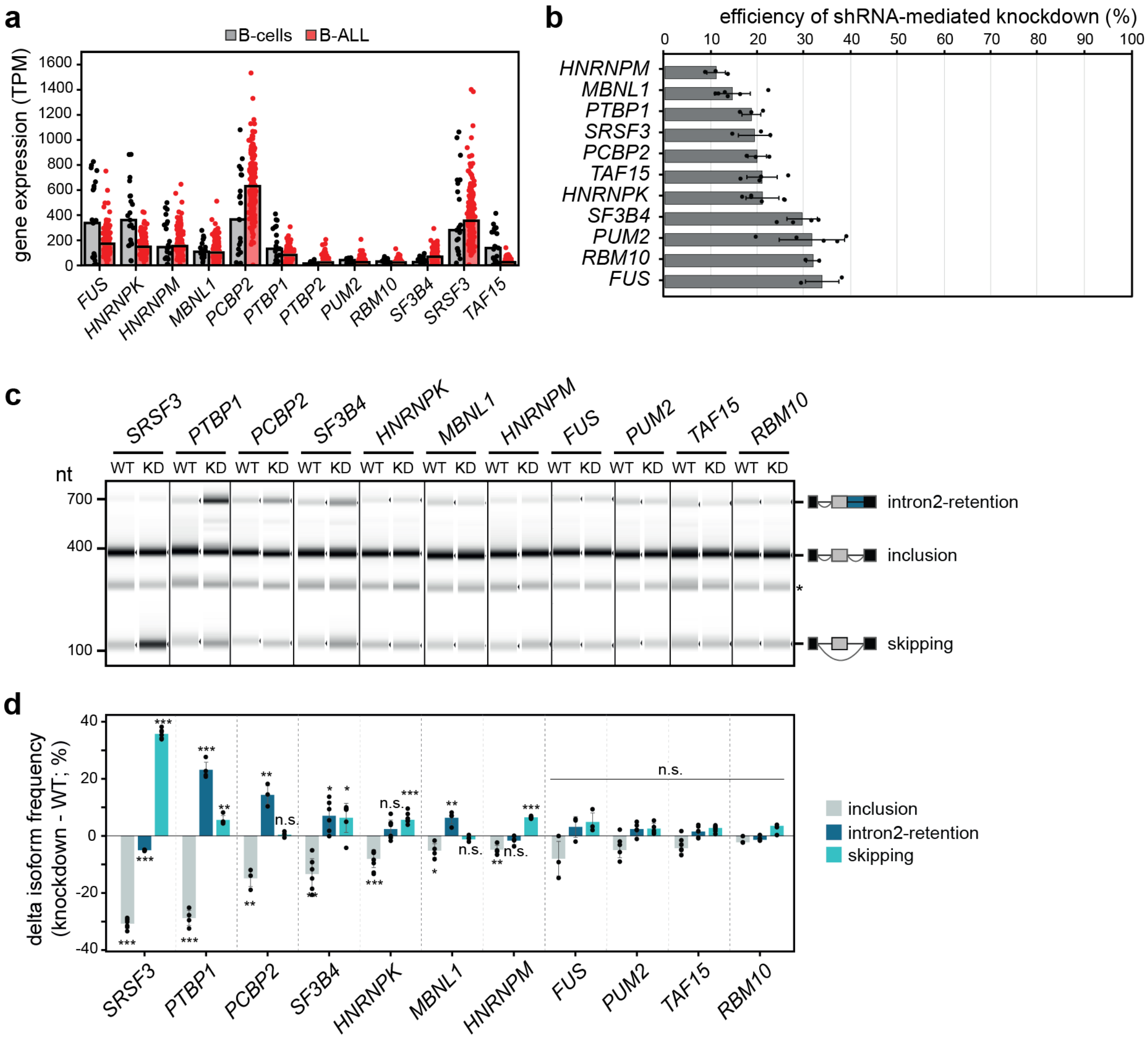

Supplementary Figure 8. Knockdown experiments show significant effects on endogenous *CD19* splicing for seven candidate RBPs. (a) The tested RBPs are expressed in patients. Barplot shows RBP mRNA levels (TPM) for normal B-cells (n = 21) and TARGET B-ALL patient samples (n = 220). **(b)** All tested RBPs are efficiently depleted upon shRNA knockdown (KD). Barplot shows mean qPCR measurements of remaining transcripts (relative to WT) for 11 candidate RBPs. Error bars indicate standard deviation of the mean (s.d.m.), n = 3 replicates. **(c, d)** Seven RBP knockdowns significantly affect *CD19* alternative splicing. Semiquantitative RT-PCR was performed to detect isoforms generated from exons 1-3 of the endogenous *CD19* gene. Gel-like representation (c), with major isoforms indicated on the right, and quantification (d), as difference in isoform frequency compared to WT, are shown. Error bars indicate s.d.m., n = 3 replicates. * *P* value < 0.05, ** *P* value < 0.01, *** *P* value < 0.01, n.s., not significant, two-sided Student's *t*-test. Source data including *P* values are provided as a Source Data file.

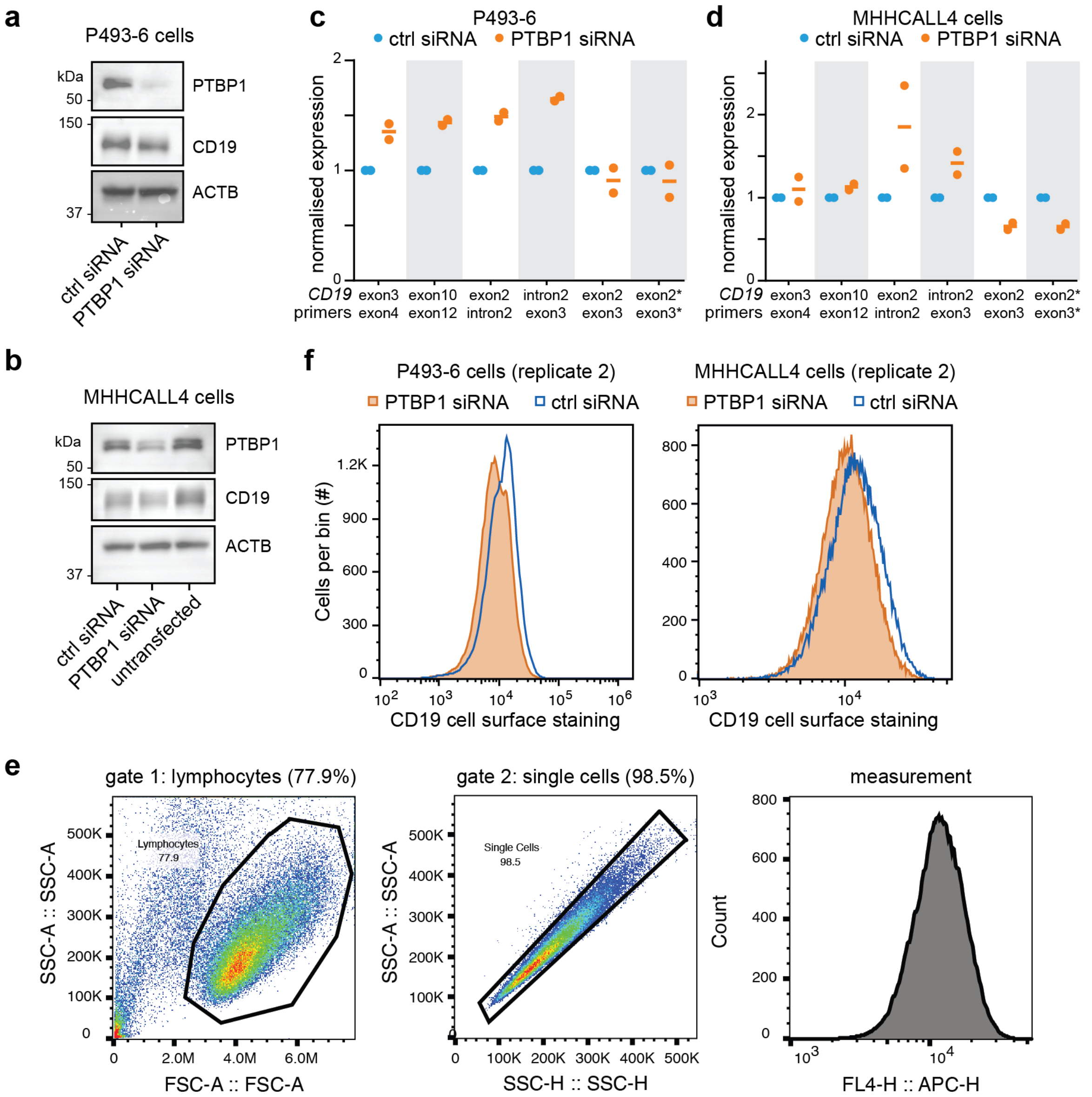

Supplementary Figure 9. PTBP1 regulates CD19 protein surface expression. (a, b) Western blot analysis shows reduced PTBP1 and CD19 protein expression upon siRNA-mediated *PTBP1* knockdown in P493-6 (a) and MHHCALL4 (b) cells, two human B-cell lines derived from immortalised lymphocytes and B-ALL tumour cells, respectively (n = 2, exemplary data are shown). Actin B (ACTB) served as loading control. Uncropped images of the gels are provided in Supplementary Figure 10. **(c, d)** *CD19* intron 2 retention is increased upon *PTBP1* knockdown in P493-6 (c) and MHHCALL4 (d) cells. Barplots show qPCR quantification of different exon-exon and exon-intron junctions as indicated below. Samples were normalised to *GAPDH* mRNA and the non-targeting control siRNA condition. Error bars indicate standard deviation (n = 2 biological replicates). **(e)** Gating strategy for the flow cytometry analysis of CD19 surface protein exposure. The first gate was set for the cell population, the next gate for singlets and finally, immunostaining of CD19 surface protein was measured in the allophycocyanin (APC) channel. **(f)** CD19 cell surface staining is reduced upon *PTBP1* knockdown in P493-6 (left panel; replicate 2) and MHHCALL4 (right panel; replicate 2) cells. Distributions of CD19 surface protein, as measured in 45-50x10^3 cells per replicate by CD19 antibody staining and flow cytometry, in cells transfected with PTBP1 siRNA (orange) or non-targeting control siRNA (blue). The results for replicate 1 are shown in Figure 6d, e.

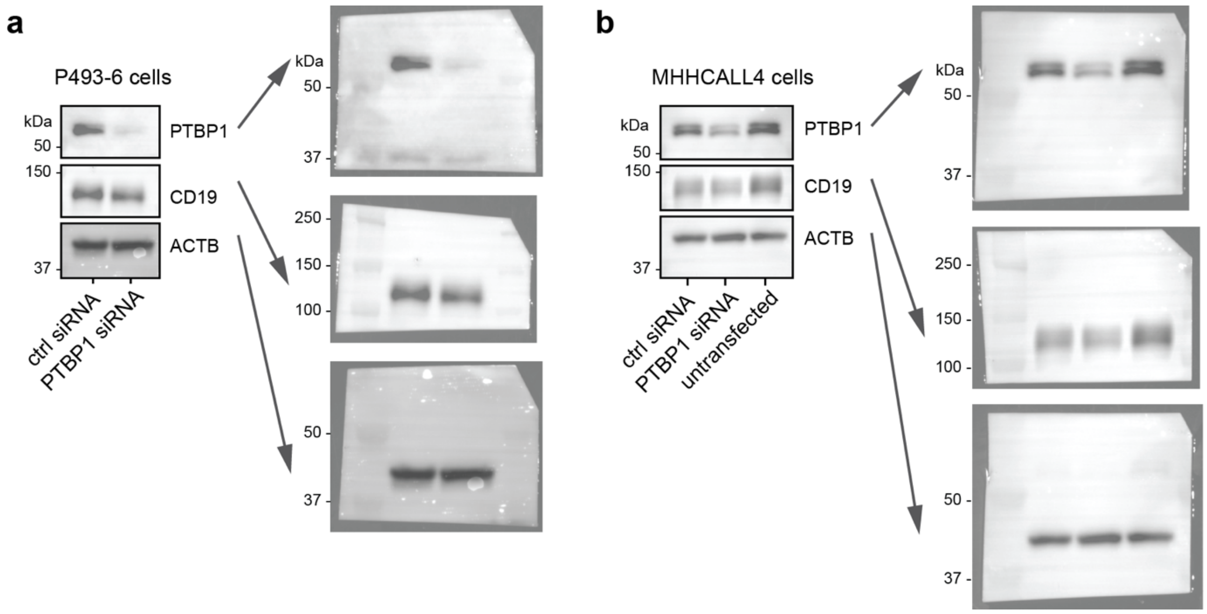

Supplementary Figure 10. Uncropped images for Western blots in Supplementary Figure 9a, b. Western blot analysis shows reduced PTBP1 and CD19 protein expression upon siRNA-mediated *PTBP1* knockdown in P493-6 and MHHCALL4 cells. Actin B (ACTB) served as loading control.

Supplementary Tables

Supplementary Table 1. Mutations from relapsed B-ALL patients reported in Orlando et al. that were tested in the *CD19* minigene splicing reporter. Patient IDs are given as reported in Orlando et al.[2]. Note that for patient #14, two separate minigene variants were tested (#14.1 and #14.2), and that #14.2 is a combination of two adjacent mutations reported in patient #14, namely c.509A>AGTGG and c.510GCCTC>GTGGGGGAG.

patient ID	mutation	genomic coordinate (hg38)	position in minigene	reference allele (REF)	alternative allele (ALT)
#2	c.259G>GGGGGC	chr16:28932516	646	G	GGGGGC
#4	c.517TGTCTCCCACCG>T	chr16:28933072	1202	TGTCTCCCACCG	T
#5	c.269AGATGGGG>A	chr16:28932526	656	AGATGGGG	A
#8	c.265CA>C	chr16:28932522	652	CA	C
#11	c.264TCAACAGATGGGGGGCTTCTACCTGTGC>T	chr16:28932521	651	TCAACAGATGGGGGGCTTCTACCTGTGC	T
#13	c.421T>TC	chr16:28932976	1106	T	TC
#14.1	c.297GGGGC>G	chr16:28932554	684	GGGGC	G
#14.2	c.510AGCCTC>AGTGGGGGAG	chr16:28933065	1195	AGCCTC	AGTGGGGGAG
#15	c.271ATGGGGGGCTTCTACCTGTGCCAGCCGGGGCCC>AAGACGT	chr16:28932528	658	ATGGGGGGCTTCTACCTGTGCCAGCCGGGGCCC	AAGACGT

Supplementary Table 2. Oligonucleotides used to clone the different shRNA sequence carrying vectors in this study. Oligonucleotides were purchased from Integrated DNA Technologies.

shRNA_FUS	TGCTGTTGACAGTGAGCGCACAGGATAATTCAGACAACAATAGTGAAGCCACAGATGTATTGTTGTCTGAATTATCCTGTTTGCCTACTGCCTCGGA
shRNA_HNRNPK	TGCTGTTGACAGTGAGCGACGAGTTGAGGCTGTTGATTCATAGTGAAGCCACAGATGTATGAATCAACAGCCTCAACTCGCTGCCTACTGCCTCGGA
shRNA_HNRNPM	TGCTGTTGACAGTGAGCGAAGCAGACATTCTTGAAGATAATAGTGAAGCCACAGATGTATTATCTTCAAGAATGTCTGCTCTGCCTACTGCCTCGGA
shRNA_MBNL1	TGCTGTTGACAGTGAGCGCCAGCACAATGATTGACACCAATAGTGAAGCCACAGATGTATTGGTGTCAATCATTGTGCTGTTGCCTACTGCCTCGGA
shRNA_PCBP2	TGCTGTTGACAGTGAGCGCTCCATCATTGAGTGTGTCAAATAGTGAAGCCACAGATGTATTTGACACACTCAATGATGGATTGCCTACTGCCTCGGA
shRNA_PTBP1	TGCTGTTGACAGTGAGCGCTAGCAAGATGATACAATGGTATAGTGAAGCCACAGATGTATACCATTGTATCATCTTGCTATTGCCTACTGCCTCGGA
shRNA_PUM2	TGCTGTTGACAGTGAGCGCAACATAGTTGTTGACTGTTAATAGTGAAGCCACAGATGTATTAACAGTCAACAACTATGTTATGCCTACTGCCTCGGA
shRNA_RBM10	TGCTGTTGACAGTGAGCGCCGGCAAGACCATCAATGTTGATAGTGAAGCCACAGATGTATCAACATTGATGGTCTTGCCGTTGCCTACTGCCTCGGA
shRNA_SF3B4	TGCTGTTGACAGTGAGCGCTGCCTTCAAGAAGGACTCCAATAGTGAAGCCACAGATGTATTGGAGTCCTTCTTGAAGGCATTGCCTACTGCCTCGGA
shRNA_SRSF3	TGCTGTTGACAGTGAGCGCTAAGATGTTTTAGCTGTTCAATAGTGAAGCCACAGATGTATTGAACAGCTAAAACATCTTAATGCCTACTGCCTCGGA
shRNA_TAF15	TGCTGTTGACAGTGAGCGATCAGGCTATGATCAACATCAATAGTGAAGCCACAGATGTATTGATGTTGATCATAGCCTGACTGCCTACTGCCTCGGA

Supplementary Table 3. qPCR oligonucleotide pairs used in this study. Oligonucleotides were purchased from Sigma-Aldrich.

	Forward primer	Reverse primer
qPCR_FUS	AAGGCCTGGGTGAGAATGTT	GGCTGTCCCGTTTTCTTGTT
qPCR_HNRNPK	GCGAGTTGAGGCTGTTGATT	TCAGTGGAATGAGGACAGCA
qPCR_HNRNPM	GTCAAGGGGATGTGCTGTTG	TCCGCTCAGACTATGCTTGT
qPCR_MBNL1	CGGTTTGCTCATCCTGCTGA	TTTGCACTTTTCCCGAGAGC
qPCR_PCBP2	CCAGCTCTCCGGTCATCTTT	CTGGTGCAGCTTGGTCAAAT
qPCR_PTBP1	CGAGATGAACACGGAGGAGG	CTGGATGTAGATGGGCTGGC
qPCR_PUM2	TCAGCGTCCTCTTACTCCCA	CCAGTAGCAAGACCCTGACC
qPCR_RBM10	TGTTCCCGACGTCTCTACCT	TCTCCCCATCCCAGTACAGG
qPCR_SF3B4	GAACGACTTCTGGCAGCTCA	CACAGGATTGGGAGCAGAGG
qPCR_SRSF3	CCCGGCTTTGCTTTTGTTGA	TTCCACTCTTACACGGCAGC
qPCR_TAF15	GGTCACAGGGAGGAGGTAGA	CAGCATCTGTTCTGGGTCCA
qPCR_CD19_E3E4	TGAGATCTGGGAGGGAGAG	ATCGTCCTTCAGCTCTAGGC
qPCR_CD19_E10E12	TCCTTCTCCAACGCTGAGTC	GAAGTCCATTGTCCTGGCGA
qPCR_CD19_e2i2	TGGCTGGACAGTCAATGTG	TCTCTCCAGCTCCATTGTGG
qPCR_CD19_i2e3	TCAGTATGAGCTGCTTCCTGTCC	AGCTCCCCTGGGAAGAGACC
qPCR_CD19_E2E3_1	AGGCCTGGGAATCCACATGA	GGAACAGCTCCCCGCTG
qPCR_CD19_E2E3_2	AGTCCCCGCTTAAACCCTTC	AGCTCCCCGCTGCCC
qPCR_GAPDH	ATGGGGAAGGTGAAGGTCG	GGGGTCATTGATGGCAACAATA
qPCR_ACTB	AGCATCCCCCAAAGTTCAC	AAGGGACTTCCTGTAACAACG

Supplementary References

1. Vaquero-Garcia, J. *et al.* A new view of transcriptome complexity and regulation through the lens of local splicing variations. *Elife* **5**, e11752 (2016).
2. Orlando, E. J. *et al.* Genetic mechanisms of target antigen loss in CAR19 therapy of acute lymphoblastic leukemia. *Nat Med* **24**, 1504-1506 (2018).
3. Chaisson, M. J. & Tesler, G. Mapping single molecule sequencing reads using basic local alignment with successive refinement (BLASR): application and theory. *BMC Bioinformatics* **13**, 238 (2012).
4. McKenna, A. *et al.* The Genome Analysis Toolkit: a MapReduce framework for analyzing next-generation DNA sequencing data. *Genome Res* **20**, 1297-1303 (2010).
5. Bolger, A. M., Lohse, M. & Usadel, B. Trimmomatic: a flexible trimmer for Illumina sequence data. *Bioinformatics* **30**, 2114-2120 (2014).
6. Martin, M. Cutadapt removes adapter sequences from high-throughput sequencing reads. *EMBnet.journal* **17**, 10-12 (2011).
7. Dobin, A. *et al.* STAR: ultrafast universal RNA-seq aligner. *Bioinformatics* **29**, 15-21 (2013).
8. Jaganathan, K. *et al.* Predicting Splicing from Primary Sequence with Deep Learning. *Cell* **176**, 535-548 e524 (2019).
9. Yeo, G. & Burge, C. B. Maximum entropy modeling of short sequence motifs with applications to RNA splicing signals. *J Comput Biol* **11**, 377-394 (2004).
10. Giudice, G., Sanchez-Cabo, F., Torroja, C. & Lara-Pezzi, E. ATtRACT-a database of RNA-binding proteins and associated motifs. *Database (Oxford)* **2016** (2016).
11. Benoit Bouvrette, L. P., Bovaird, S., Blanchette, M. & Lecuyer, E. oRNAment: a database of putative RNA binding protein target sites in the transcriptomes of model species. *Nucleic Acids Res* **48**, D166-D173 (2020).
12. Ghanbari, M. & Ohler, U. Deep neural networks for interpreting RNA-binding protein target preferences. *Genome Res* **30**, 214-226 (2020).
13. Gu, Z. *et al.* PAX5-driven subtypes of B-progenitor acute lymphoblastic leukemia. *Nat Genet* **51**, 296-307 (2019).
14. Alexander, T. B. *et al.* The genetic basis and cell of origin of mixed phenotype acute leukaemia. *Nature* **562**, 373-379 (2018).
15. Barretina, J. *et al.* The Cancer Cell Line Encyclopedia enables predictive modelling of anticancer drug sensitivity. *Nature* **483**, 603-607 (2012).
