# Supplementary material for: High-throughput mutagenesis identifies mutations and RNA-binding proteins controlling CD19 splicing and CART-19 therapy resistance
Source: Nat Commun. 2022 Sep 22;13:5570. doi: 10.1038/s41467-022-31818-y (PMC9500061; doi:10.1038/s41467-022-31818-y)
Supplement: Supplementary file 3 — Description of Additional Supplementary Files [file 41467_2022_31818_MOESM3_ESM.pdf]

**Supplementary Data 1. Quantification of splicing isoforms for all minigene variants in the library.** For each minigene variant, the 15-nt barcode sequence is shown together with the contained mutations, with multiple mutations separated by commas. The total number of reads per minigene variant and their distribution among the 101 isoforms are given for RNA-seq replicates 1 and 2 from NALM-6 cells. Isoform notation (219 475) indicates a splice junction that removed the region from nucleotides 219 to 475. The five major isoforms are *CD19* exon 2 inclusion (219 475)(743 1040), skipping (219 1040), intron2-retention (219 475), alt-exon2 (219 657)(743 1040) and alt-exon3 (219 475)(743 1073). In total, we detected splicing isoforms for 9,671 minigene variants in replicate 1 and for 9,372 minigene variants in replicate 2, including 9,321 minigene variants that were present in both replicates.

**Supplementary Data 2. List of detected isoforms from the *CD19* minigene.** A total of 101 isoforms reached a relative frequency of at least 5% in at least one minigene variant, including the five major isoforms inclusion, skipping, intron2-retention, alt-exon2 and alt-exon3 (>5% in WT) as well as 96 cryptic isoforms. For each isoform, the assigned name or number is shown together with the isoform specification. Isoform notation (219 475) indicates a splice junction that removed the region from nucleotides 219 to 475. With respect to the predicted impact on the encoded CD19 protein, the number of premature stop codons (PTCs), the frame (in-frame or out-of-frame) and the resulting coding potential (coding or non-coding) are reported. With respect to an isoform's relative abundance, the average isoform frequency in the library and the maximal isoform frequency in an individual minigene are given. For the 38 cryptic isoforms that are associated with a specific mutation (prevalence score > 0.25), the respective mutations are provided together with their prevalence score and genomic coordinate (hg38). Notation G475T indicates that G in position 475 was mutated to T.

**Supplementary Data 3. Single mutation effects predicted by the mathematical model.** Worksheet "Mutation effects" provides the model estimates of splice isoform frequencies (in %) and average delta frequency (compared to WT) in replicates (rep) 1 and 2 in response to individual mutations (single nucleotide variants, SNV; insertions or deletions, INDEL) in NALM-6 cells. Notation G475T indicates that G in position 475 was mutated to T. Individual entries are given for each affected isoform. Isoform notation (219 475) indicates a splice junction that removed the region from nucleotides 219 to 475. The five major isoforms are *CD19* exon 2 inclusion (219 475)(743 1040), skipping (219 1040), intron2-retention (219 475), alt-exon2 (219 657)(743 1040) and alt-exon3 (219 475)(743 1073). Worksheet "WT statistics" provides the mean, standard deviation (sd) and median of measured splice isoform frequencies (in %) for the five major isoforms as well as the sum of 96 cryptic isoforms ("other"). Isoform frequencies were measured for 195 and 194 WT minigenes in the two replicates. Worksheet "19 tested mutations" provides information on the 19 point mutations that were selected for targeted validation experiments (Figure 3e, f, Supplementary Figure 5), including the predicted mutation effects on the major isoforms in presence of the baseline mutation G742C (see Methods). G748C* is a minigene containing G748C but lacking G742C. For two previously reported single nucleotide variants, the table also summarises the information from the databases ENSEMBL [16] (v104), gnomAD [17] (v3.1), ClinVar [18] (accessed 09/2021).

**Supplementary Data 4. Overlapping single nucleotide variants (SNVs) and cancer-related mutations.** Worksheet "Annotated splic-affect variants" contains 32 SNVs (from ENSEMBL [16] v104, gnomAD [17] v3.1 and ClinVar [18] accessed 09/2021) and cancer-related variants (obtained from COSMIC [19] v94) that overlap with splicing-affecting mutations and mutations with a prevalence score > 0.25 in our screen. Notation A950G indicates that A in position 950 was mutated to G. For variants present in the database dbSNP [20], the respective ID is also included. REF and ALT refer to the reference and alternative allele. Worksheet "All annotated variants" summarises information on 830 variants from multiple databases (ENSEMBL [16] v104, gnomAD [17] v3.1, ClinVar [18] accessed 09/2021, COSMIC [19] v94 and TARGET B-ALL accessed 11/18/2021). POS is the genomic coordinate of the mutation on chromosome 16 according to the human genome version hg38. REF and ALT refer to the reference and alternative allele.

**Supplementary Data 5. SpliceAI predictions for the complete *CD19* gene.** Worksheet "All predictions" provides information on the SpliceAI predictions for all 22,158 mutations along the complete *CD19* gene. Worksheet "Annotated SNVs with score > 0.2" highlights 37 mutations that overlap with a reported single nucleotide variant (SNV, see below) and for which SpliceAI predicts the gain or loss of a 5' (donor) or 3' (acceptor) splice site with SpliceAI score > 0.2 (recommended cutoff; Supplementary Figure 5c). POS is the genomic coordinate of the mutation on chromosome 16

according to the human genome version hg38. REF and ALT refer to the reference and alternative allele. Variant_set and Variant_ID are given if the mutation corresponds to an annotated SNV (from ENSEMBL [16] v104, gnomAD [17] v3.1 and ClinVar [18] accessed 09/2021) or a cancer-related variant (obtained from COSMIC [19] v94 and TARGET B-ALL). POS_DG/DL/AG/AL and DS_DG/DL/AG/AL indicate the genomic coordinates (POS) and SpliceAI score (delta score, DS) of donor gain (DG) and loss (DL) and acceptor gain (AG) and loss (AL), respectively.

**Supplementary Data 6. Predicted RBP binding sites in the region of the *CD19* minigene.** Worksheet “Binding sites” reports *in silico* predictions by ATtRACT [10] and oRNAment [11], providing the source tool, start and end and width (relative to the *CD19* minigene), predicted RNA-binding protein (RBP) and whether the binding site overlaps with splicing-affecting mutations from our screen (see Methods). Worksheet “DeepRiPe mutations” reports all mutations predicted by DeepRiPe [12] to change RBP binding (i.e., with an absolute delta score > 0.1), including RBP, mutation, DeepRiPe score and set as well as whether the mutation overlaps with a splicing-affecting mutation from our screen and if so, for which isoform. Positive and negative delta scores refer to a predicted increase or reduction in RBP binding, respectively. Set refers to the DeepRiPe model that was trained for a given RBP using PAR-CLIP or ENCODE eCLIP data from HepG2 or K562 cells (see [12] for details).
