# Supplementary material for: High-throughput mutagenesis identifies mutations and RNA-binding proteins controlling CD19 splicing and CART-19 therapy resistance
Source: Nat Commun. 2022 Sep 22;13:5570. doi: 10.1038/s41467-022-31818-y (PMC9500061; doi:10.1038/s41467-022-31818-y)
Supplement: Supplementary file 10 — Reporting Summary [file 41467_2022_31818_MOESM10_ESM.pdf]

nature portfolio

Corresponding author(s): Dr. Katharina Zarnack

Last updated by author(s): 26.06.2022

Reporting Summary

Nature Portfolio wishes to improve the reproducibility of the work that we publish. This form provides structure for consistency and transparency in reporting. For further information on Nature Portfolio policies, see our Editorial Policies and the Editorial Policy Checklist.

Statistics

For all statistical analyses, confirm that the following items are present in the figure legend, table legend, main text, or Methods section.

n/a	Confirmed	
[ ]	[x]	The exact sample size (n) for each experimental group/condition, given as a discrete number and unit of measurement
[ ]	[x]	A statement on whether measurements were taken from distinct samples or whether the same sample was measured repeatedly
[ ]	[x]	The statistical test(s) used AND whether they are one- or two-sided<br>*Only common tests should be described solely by name; describe more complex techniques in the Methods section.*
[ ]	[x]	A description of all covariates tested
[ ]	[x]	A description of any assumptions or corrections, such as tests of normality and adjustment for multiple comparisons
[ ]	[x]	A full description of the statistical parameters including central tendency (e.g. means) or other basic estimates (e.g. regression coefficient) AND variation (e.g. standard deviation) or associated estimates of uncertainty (e.g. confidence intervals)
[ ]	[x]	For null hypothesis testing, the test statistic (e.g. F, t, r) with confidence intervals, effect sizes, degrees of freedom and P value noted<br>*Give P values as exact values whenever suitable.*
[x]	[ ]	For Bayesian analysis, information on the choice of priors and Markov chain Monte Carlo settings
[x]	[ ]	For hierarchical and complex designs, identification of the appropriate level for tests and full reporting of outcomes
[ ]	[x]	Estimates of effect sizes (e.g. Cohen's d, Pearson's r), indicating how they were calculated

Our web collection on statistics for biologists contains articles on many of the points above.

Software and code

Policy information about availability of computer code

Data collection

The following software was used:
STAR (v2.6.1)
ASpli (version 1.12.1)
BiomaRt (version 2.4.21)
OrgDb (version 3.10.0)
edgeR (version 3.28.1)
GATK HaplotypeCaller (v4.0.10)
FastQC (v0.11.5)
Trimmomatic (v0.36)
Cutadapt (version 1.6)
SAMtools (version 1.5)
maxentpy (version 0.0.1)
SpliceAI (version 1.3.1)
FastQC (version 0.11.8)
FASTX-Toolkit (version 0.0.14)
seqtk (version 1.3)
Flexbar (version 3.4.0)
TrimGalore (version 0.6.6)
STAR (version 2.5.2a)
MAJIQ (version 2.2)
SAMtools (version 1.11)

	FlowJo (version 10.7.2)
Data analysis	All data analysis steps performed for this manuscript are described in detail in the manuscript. The scripts used to process the files are accessible under the GitHub repository located at: https://github.com/mcortes-lopez/CD19_splicing_mutagenesis.

For manuscripts utilizing custom algorithms or software that are central to the research but not yet described in published literature, software must be made available to editors and reviewers. We strongly encourage code deposition in a community repository (e.g. GitHub). See the Nature Portfolio guidelines for submitting code & software for further information.

Data

Policy information about availability of data

All manuscripts must include a data availability statement. This statement should provide the following information, where applicable:
- Accession codes, unique identifiers, or web links for publicly available datasets
- A description of any restrictions on data availability
- For clinical datasets or third party data, please ensure that the statement adheres to our policy

The sequencing data generated in this study have been deposited in the Gene Expression Omnibus (GEO) database under accession code GSE182894 [https://www.ncbi.nlm.nih.gov/geo/query/acc.cgi?acc=GSE182894]. The collection consists of the PacBio DNA-seq libraries (GSE182892) [https://www.ncbi.nlm.nih.gov/geo/query/acc.cgi?acc=GSE182891], the Illumina RNA-seq libraries (GSE182892) [https://www.ncbi.nlm.nih.gov/geo/query/acc.cgi?acc=GSE182892] and the PTBP1 iCLIP2 libraries in NALM-6 cells (GSE182893) [https://www.ncbi.nlm.nih.gov/geo/query/acc.cgi?acc=GSE182893].
The results published here are in whole or part based upon data generated by the Therapeutically Applicable Research to Generate Effective Treatments (https://ocg.cancer.gov/programs/target) initiative, phs000218. The data used for this analysis are available at https://portal.gdc.cancer.gov/projects.
The remaining data are available within the Article, Supplementary Information or Source Data files.

Field-specific reporting

Please select the one below that is the best fit for your research. If you are not sure, read the appropriate sections before making your selection.

☒ Life sciences ☐ Behavioural & social sciences ☐ Ecological, evolutionary & environmental sciences

For a reference copy of the document with all sections, see nature.com/documents/nr-reporting-summary-flat.pdf

Life sciences study design

All studies must disclose on these points even when the disclosure is negative.

Sample size	Sample sizes were chosen based on accepted practices in the filed and stated in each figure or figure legend, with 2 or more biological replicates as indicated.
Data exclusions	We excluded one patient (patient #17) from Orlando et al. after visual inspection indicating that the submitted data in fact corresponded to DNA-seq rather than RNA-seq data.
Replication	All experiments were performed in 2 or more replicates as indicated in the figure legends and methods.
Randomization	Samples were collected to study groups by genome type and/or treatment condition. No randomisation was applied.
Blinding	Blinding was not relevant due to the objective readouts (e.g. RNA-seq, DNA-seq, RT-PCR) and investigators were not blinded.

Reporting for specific materials, systems and methods

We require information from authors about some types of materials, experimental systems and methods used in many studies. Here, indicate whether each material, system or method listed is relevant to your study. If you are not sure if a list item applies to your research, read the appropriate section before selecting a response.

Materials & experimental systems

n/a	Involved in the study	
☐	☒	Antibodies
☐	☒	Eukaryotic cell lines
☒	☐	Palaeontology and archaeology
☒	☐	Animals and other organisms
☒	☐	Human research participants
☒	☐	Clinical data
☒	☐	Dual use research of concern

Methods

n/a	Involved in the study	
☒	☐	ChIP-seq
☐	☒	Flow cytometry
☒	☐	MRI-based neuroimaging

Antibodies

Antibodies used	The following antibodies were used in this study:<br>anti-PTBP1 antibody (Santa Cruz, sc-56701)<br>anti-human CD19 antibody (982406, BioLegend)<br>β-Actin (8H10D10, Cell Signaling)
Validation	The used antibodies were obtained from commercial vendors which ensure the quality of the antibody.

Eukaryotic cell lines

Policy information about cell lines

Cell line source(s)	NALM-6 cells were obtained from ATCC. HEK293 and HEK293T cells were obtained from DSMZ.
Authentication	None of the cell lines used were authenticated.
Mycoplasma contamination	All cells were routinely for mycoplasma infection and always tested negative.
Commonly misidentified lines<br>(See ICLAC register)	No commonly misidentified lines were used in this study.

Flow Cytometry

Plots

Confirm that:

- [x] The axis labels state the marker and fluorochrome used (e.g. CD4-FITC).
- [x] The axis scales are clearly visible. Include numbers along axes only for bottom left plot of group (a 'group' is an analysis of identical markers).
- [x] All plots are contour plots with outliers or pseudocolor plots.
- [x] A numerical value for number of cells or percentage (with statistics) is provided.

Methodology

Sample preparation	1x105 cells were resuspended in 50 µl of PBS, 20% FBS, 1 mM EDTA and 2.5 µl of Human TruStain FcX blocking (422302, BioLegend) and incubated for 20 min. After blocking, 2.5 µl of APC anti-human CD19 antibody (982406, BioLegend) was added to the cells and incubated for 30 min. Cells were washed twice with PBS, 20% FBS, 1 mM EDTA and the CD19 staining was measured using the BD Accuri C6 Plus Flow Cytometer instrument (BD Biosciences).
Instrument	BD Accuri C6 Plus Flow Cytometer instrument (BD Biosciences)
Software	Flow cytometry data was analyzed with FlowJo_v.10.7.2 software.
Cell population abundance	No cell sorting
Gating strategy	For gating, we used the stained and unstained parental cells to set the gate for the correct cell population. Also, these same controls were used to establish the cutoffs for the CD19 staining.

- [x] Tick this box to confirm that a figure exemplifying the gating strategy is provided in the Supplementary Information.
