# Supplementary material for: High-throughput mutagenesis identifies mutations and RNA-binding proteins controlling CD19 splicing and CART-19 therapy resistance
Source: Nat Commun. 2022 Sep 22;13:5570. doi: 10.1038/s41467-022-31818-y (PMC9500061; doi:10.1038/s41467-022-31818-y)
Supplement: Supplementary file 11 — Source Data file [file 41467_2022_31818_MOESM11_ESM.zip › Source Data/Source Data mutation effect profiles.pdf]

Data S1. Single mutation effects on the major isoforms from the CD19 minigene in NALM−6 cells.

For each isoform, the y−axis shows the isoform frequency (mean of two biological replicates) resulting from each individual mutation in a given
position along the y−axis. Each dot represents one mutation, with colours indicating the inserted nucleotide (green, mutation to A; blue, to C; yellow, to G; red, to T).
Splicing−effective mutations are shown as filled circles and non−effective mutations as open circles. Dashed lines indicate the median isoform frequency of the WT minigenes (black)
± 2 standard deviations (grey). The shown isoforms are CD19 exon 2 inclusion, skipping, intron2−retention, alt−exon2 and alt−exon3 as well as the sum of 96 cryptic isoforms ("other").

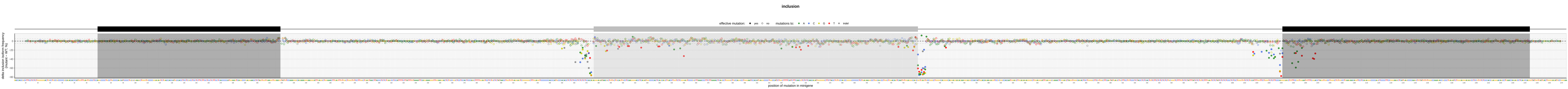

skipping

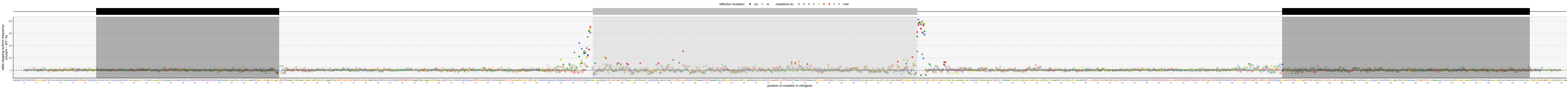

effective mutation: yes no
mutations to: A C G T indel
delta skipping isoform frequency (mutant − WT; %)
position of mutation in minigene

intron2 retention

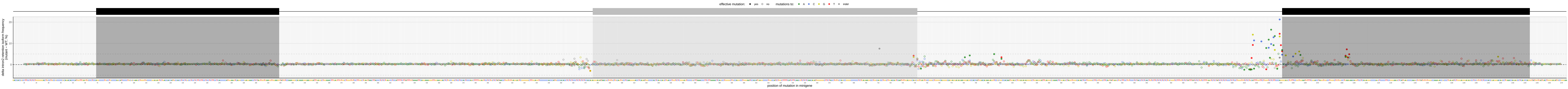

alt−exon2

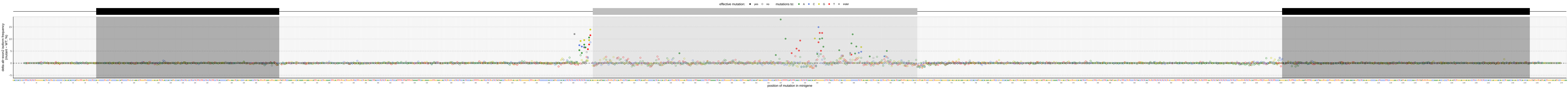

effective mutation: yes no
mutations to: A C G T indel
delta alt−exon2 isoform frequency
(mutant − WT; %)
15
10
5
0
−5
position of mutation in minigene

alt−exon3

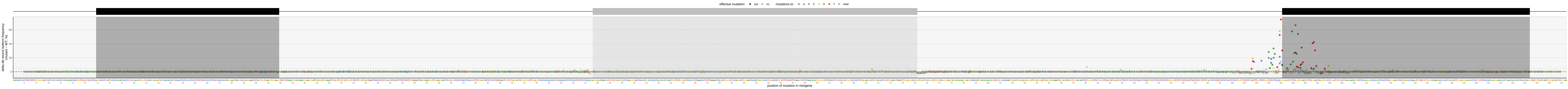

effective mutation:
yes
no
mutations to:
A
C
G
T
indel
delta alt−exon3 isoform frequency (mutant − WT; %)
60
40
20
0
position of mutation in minigene

other

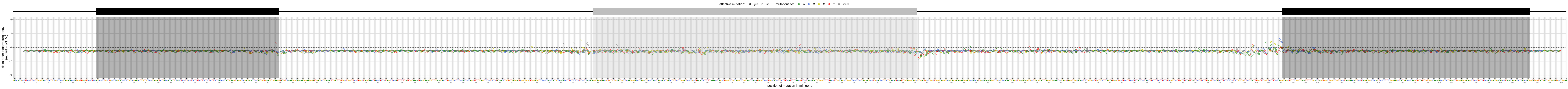
effective mutation: yes no
mutations to: A C G T indel
delta other isoform frequency (mutant – WT; %)
5
3
0
−2
−5
position of mutation in minigene
